# A Federated Learning Multi-Task Scheduling Mechanism Based on Trusted Computing Sandbox

**DOI:** 10.3390/s23042093

**Published:** 2023-02-13

**Authors:** Hongbin Liu, Han Zhou, Hao Chen, Yong Yan, Jianping Huang, Ao Xiong, Shaojie Yang, Jiewei Chen, Shaoyong Guo

**Affiliations:** 1 State Grid Corporation of China, Beijing 100031, China; 2 State Key Laboratory of Network and Switching Technology, Beijing University of Posts and Telecommunications, Beijing 100876, China; 3 State Grid Zhejiang Electric Power Co., LTD., Hangzhou 310007, China

**Keywords:** blockchain, computing sandbox, data privacy, resource scheduling, deep reinforcement learning

## Abstract

At present, some studies have combined federated learning with blockchain, so that participants can conduct federated learning tasks under decentralized conditions, sharing and aggregating model parameters. However, these schemes do not take into account the trusted supervision of federated learning and the case of malicious node attacks. This paper introduces the concept of a trusted computing sandbox to solve this problem. A federated learning multi-task scheduling mechanism based on a trusted computing sandbox is designed and a decentralized trusted computing sandbox composed of computing resources provided by each participant is constructed as a state channel. The training process of the model is carried out in the channel and the malicious behavior is supervised by the smart contract, ensuring the data privacy of the participant node and the reliability of the calculation during the training process. In addition, considering the resource heterogeneity of participant nodes, the deep reinforcement learning method was used in this paper to solve the resource scheduling optimization problem in the process of constructing the state channel. The proposed algorithm aims to minimize the completion time of the system and improve the efficiency of the system while meeting the requirements of tasks on service quality as much as possible. Experimental results show that the proposed algorithm has better performance than the traditional heuristic algorithm and meta-heuristic algorithm.

## 1. Introduction

In the past decade, the global data flow has been growing at an unprecedented speed, and the value behind the data has been paid more and more attention. Intelligent applications supported by massive data will further promote the coordinated development of power enterprises and financial services in the industrial chain. However, due to the problem of data isolation between different departments and systems, the cost of cross-domain data circulation is high, and there is a risk of privacy disclosure, which hinders the full release of the potential value of data.

Traditional centralized financial services in the industrial chain generally have problems such as high cost of verification, incomplete information, difficulty in the supervision of repeated financing, fake financing, and increased financing costs. As a distributed ledger technology [1], blockchain has emerged as a solution to the problem of secure data sharing, providing participants with high-quality data and secure data sharing. At present, some researchers combine industry chain finance with blockchain and integrate blockchain technology into complex business scenarios of industry chain finance. This also brings the problem of the security of the industrial chain financial data privacy in the multi-party data-sharing environment. Although blockchain-based multi-party data sharing can make the data open and transparent, it cannot guarantee the data privacy security of users. How to protect data privacy in the case of data sharing is a hot topic in current research.

Federated learning (FL), as a new data value-sharing method, aims to solve the problem of data isolation and protect data privacy. Different from traditional machine learning, participants in federated learning do not need to upload their local data but only need to upload parameters and models trained with local data to the central server, and then, the central server will aggregate the global model, thus protecting the privacy and security of their data.

Traditional federated learning relies on a single central server. If this weak central server fails or is attacked, the global model will be inaccurate and even the whole federated learning process will be hindered. In addition, the inability to guarantee whether the central server itself has malicious behavior means that the data privacy and security of each participant will not be guaranteed. The combination of blockchain and federated learning can well protect the privacy of user data and share data value in a decentralized scenario. There have been many studies that use the characteristics of blockchain to replace the central server. At the same time, the nodes with good performance are provided with corresponding rewards; thus, the nodes with better data are encouraged to participate in the training more actively. These mechanisms and schemes use blockchain technology to verify and supervise the original data and final calculated results of federated learning. Although this approach can share the value of data and protect the privacy of users to a certain extent by keeping the data local, it does not take into account whether the computation is credible in the training process of the federated learning model and the privacy leakage caused by malicious node attacks. It ignores the trust supervision in the training and aggregation process of the federated learning model. Therefore, it is a challenging task to build a trusted computing framework that ensures data privacy and security for all participants.

In this paper, to solve this problem, we design a federal learning training supervision mechanism based on state channels by introducing the concept of a computational sandbox in trusted computing. The major contributions to this work are as follows:

- A trusted regulatory framework for federated learning training transactions based on blockchain state channel is designed, and a decentralized trusted computing sandbox composed of computing resources provided by each participant is constructed. The model training process is carried out in the state channel, and the malicious behavior is supervised by a smart contract.
- Aiming at the resource heterogeneity problem of the federated learning participant node, a participant resource management method was proposed to model the resource scheduling optimization problem existing in the process of constructing the state channel. The deep reinforcement learning (DRL) method was used to solve the proposed optimization problem, minimizing the maximum completion time of the system under the condition of meeting the requirements of tasks on service quality.
- We compare the resource-scheduling algorithm based on DRL with the traditional heuristic and meta-heuristic algorithms through simulation experiments. The experiments show that the algorithm has better performance in meeting the requirements of tasks on service quality and reducing the maximum completion time of the system.

The rest of this paper is organized as follows. Section 2 describes the related work. Section 3 introduces the system framework and workflow of the mechanism. In Section 4, the resource-scheduling problem in the process of constructing a state channel is modeled. Section 5 presents the resource scheduling algorithm based on DRL. In Section 6, the proposed algorithm is verified by experimental simulation. Section 7 summarizes the work of the thesis.

## 2. Related Works

### 2.1. Decentralized Federated Learning Framework

Federated learning has become increasingly important for modern machine learning in data privacy-sensitive scenarios. The existing federated learning mainly adopts the network topology based on a central server [2,3,4,5]. However, in some cases, this connection method is not suitable. For example, there is no central server connecting all users; the communication cost to the central server is unbearable; the central server cannot be fully trusted.

Thus, to further protect data privacy and avoid communication bottlenecks, decentralized architectures have been proposed recently [6,7,8,9,10,11,12]. Decentralized architectures remove a central node and each node only communicates with its neighbors (trust each other) by exchanging its local model. Ref. [13] provides a systematic analysis of decentralized learning.

Blockchain is one of the most popular disruptive technologies [14]. With the characteristics of decentralization and data not easily tampered with, it can provide a high degree of guarantee for secure data collection and sharing, paving the way for emerging financial and industrial services.

Many studies have combined blockchain technology with federated learning to ensure the value of sharing data in the context of decentralization and data privacy. Ref. [15] designed a distributed multi-party secure data-sharing architecture based on blockchain authorization to transform the data-sharing problem into a machine learning problem. By sharing the data model instead of revealing the actual data, data privacy was well maintained. To balance the issues of private security and efficiency in fog computing, ref. [16] proposes a new federated learning (FL-Block) scheme based on blockchain, which allows local devices to exchange and update the global model through blockchain. Ref. [17] designed a federated learning system under the blockchain scenario, which uses the reputation mechanism to assist home appliance manufacturers to train machine learning models and predict future consumer demand and consumption behavior. However, these schemes do not guarantee the reliability and security of the training process. The trained model and parameters will be sent to other nodes for aggregation. If other nodes have malicious behaviors, personal privacy information can still be obtained by attacking FL model parameters.

### 2.2. Resource Scheduling Problem

The existing methods for solving scheduling problems include dynamic programming, probabilistic algorithm, heuristic algorithm, meta-heuristic algorithm, hybrid algorithm, and deep reinforcement learning methods.

The resource-scheduling problem is often abstracted as a target optimization problem. Traditional heuristic resource scheduling algorithms include first-come-first-serve FCFS [18], RR [19], Min–min [20], Max–min [20], etc. Metaheuristic algorithms are algorithms inspired by biological behavior and natural phenomena that imitate biological behavior, including genetic algorithm [21], particle swarm optimization algorithm [22], ant colony algorithm [23], etc. Genetic algorithm [21] applies the principle of biological evolution to obtain high-quality solutions from the search space in polynomial time, and it generates new solutions by randomly modifying better solutions. Ref. [24] proposes a hybridized monarch butterfly optimization algorithm, which is suitable for solving cloud-scheduling problems. Ref. [25] combines two optimization algorithms, namely CS (cuckoo search) and PSO (particle swarm optimization search), to reduce the completion time, cost, and deadline default rate.

In practical application, the cloud system has the following characteristics: (1) the system is large and complex, and cannot be modeled accurately; (2) the timeliness of scheduling decisions requires a high-speed scheduling algorithm; (3) randomness of tasks (or requests), including randomness of task number, arrival time and size. These characteristics pose a challenge to resource-scheduling research in cloud computing. It is difficult for a particular meta-heuristic or heuristic algorithm to fully adapt to real dynamic cloud computing systems or edge cloud computing systems. Deep reinforcement learning (DRL) is a new method of machine learning, which combines the advantages of deep neural networks and reinforcement learning and has been used to solve resource-scheduling problems of cloud computing in recent years. It has been proved to have strong advantages in many scenarios, especially in complex scenarios of cloud computing [26,27,28,29,30,31]. Ref. [32] proposed a task-scheduling framework based on Q learning. All requests are prioritized and then tasks are assigned to the virtual machine using a constantly updated policy that minimizes task response time and maximizes CPU utilization. Ref. [33] established a model based on a reinforcement learning K-mean algorithm and developed a physical resource allocation scheme to meet the service quality requirements of users. Ref. [26] proposes a deep reinforcement learning solution based on DRL to effectively solve different cloud resource management problems. The above scheme provides the model and algorithm of the network resource scheduling problem but does not consider the characteristics of federated learning and the training environment.

Aiming at the problems of model attacks of malicious nodes in the federated learning process and whether the computing process is safe and reliable, this paper introduces the concept of computing sandbox to establish the state channel in the federated learning process, ensuring the security and trust of computing in the process of model training and the supervision of malicious behaviors. To solve the container-based resource management problem under this framework, we also propose a resource scheduling strategy for federated learning tasks based on the requirements of tasks on service quality and system completion time, improving the resource utilization rate of network collaborative federated learning and improving the efficiency of the system to complete federated learning tasks.

## 3. System Model

### 3.1. System Framework Structure

**Figure 1:**
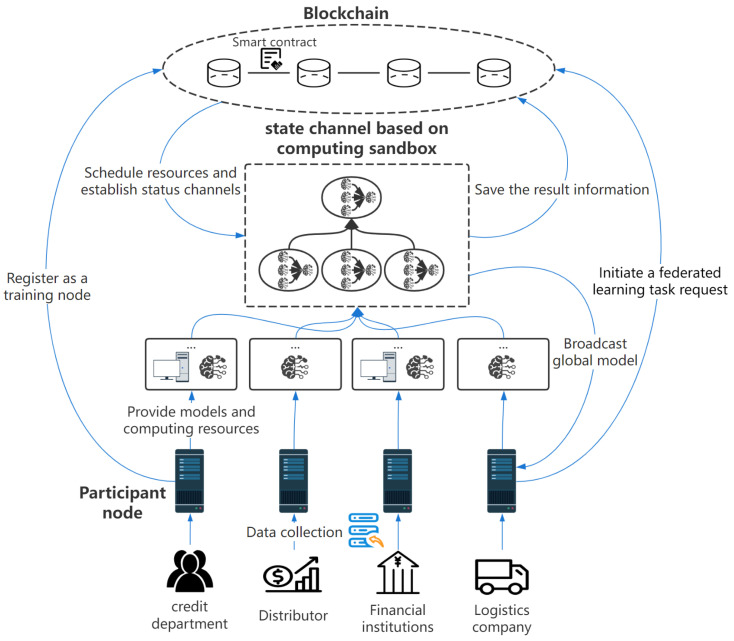
Federated Learning Training Framework Based on State Channel.

The system architecture constructed in this paper is shown in Figure 1. By introducing the concept of computational sandbox in trusted computing, a federated learning framework based on state channels is constructed in the blockchain scenario. The model parameter passing and aggregation of federated learning tasks are regulated in the computational sandbox state channel, and the occupied resources are released after the task is completed. The result is then credibly transmitted to the requester. This process supports the trusted calculation and sharing of data, which is invisible to the training participants. The whole system framework is mainly divided into three parts: participant nodes, blockchain and state channel.

The participant nodes can be either the requester or the local training node of the federated learning task. Each participant has user data and resources with training value, representing a certain industry or enterprise, such as financial institutions, credit investigation departments, etc. Participant nodes do not want to share user data with other parties but rather want to share the value of data through federated learning by co-training the global model with local data. The participant node can apply to the blockchain for registration as a training node, and it can apply to cooperate with other participant nodes to conduct federated learning tasks and train the global model.

In the traditional federated learning process, locally trained model parameters need to be sent to the central server or aggregation node, where the global model can be aggregated. In this case, the single point of attack on the aggregation node can easily cause the collapse of the entire federated learning system, local data privacy leakage, and other security problems.

To this end, we introduce a computing sandbox as a blockchain state channel. The global model aggregation of federated learning tasks and the calculation of parameters depend on the computing resources and space provided by each participant. We perform the computation in a state channel, which is regulated by the blockchain. In the process of constructing the state channel, the blockchain reasonably schedules resources to the federated learning task according to the requirements of different tasks, making the whole system work more efficiently. The scheduling strategy determines the efficiency and resource utilization of the whole system. Federated learning tasks do not own or retain allocated resources. Instead, the system dynamically allocates them based on current needs using scheduling algorithms to make full use of resources and release used resources immediately after the task is completed. The resource-scheduling problem and scheduling policy are discussed in Section 4 and Section 5.

Blockchain is used to manage the status and verification information of each participant, integrate and virtualize various resources registered by each participant, such as computing resources and storage space, and coordinate and supervise the federated learning of each participant through smart contracts. Considering that different participant nodes have different computing power and storage resources, some participant nodes do not have the ability to complete the model training task independently. By integrating the resources of all nodes and constructing state channels with reasonable allocation and scheduling, the problem of resource heterogeneity can be well coordinated and solved. When the task request arrives, the blockchain will conduct resource scheduling, reasonably allocate resources to the task, and establish the federal learning state channel. After that, the training of the federated learning task model and the transmission of parameters will be carried out in the state channel. Any malicious behavior will be supervised by all nodes and reported to the blockchain to ensure the reliable calculation of data and the security of the model training aggregation process.

To protect the privacy and security of user data, the real physical address of the state channel should not be disclosed to all participant nodes. The blockchain is responsible for masking the real physical address of the state channel from the participant nodes and only providing the virtual address and interface to the participant nodes. The federated learning task is completed in the state channel, and each participant node cannot obtain the model and parameters of other participant nodes, which means the participant node cannot attack the original user data.

### 3.2. System Work Flow

**Figure 2:**
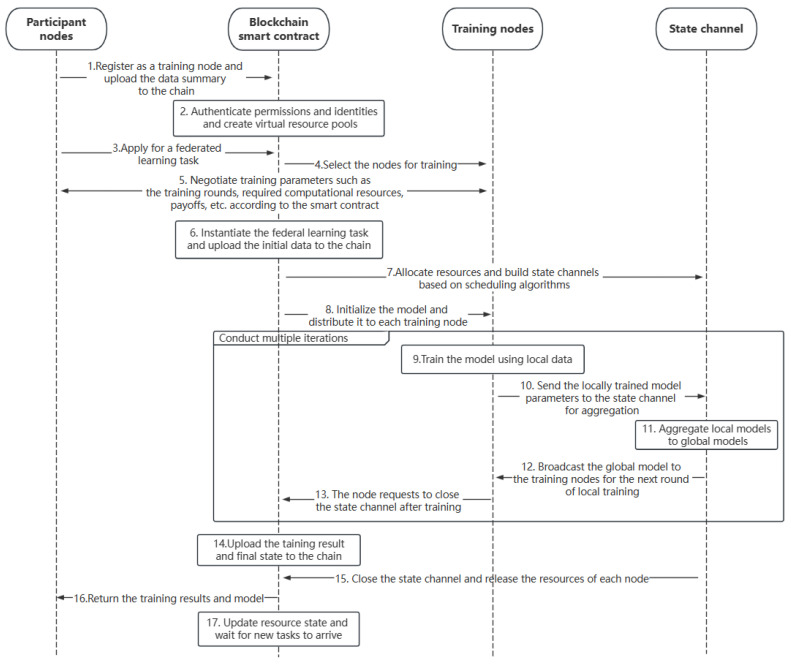
System Working Flow Chart.

As shown in Figure 2, the system workflow is mainly divided into the following steps.

In the first step, a participant who wants to instantiate a federated learning task to aggregate the global model and share the value of the data must first register with the blockchain as a training node. Participants submit identity information and describe the data they own and the resources they can provide, including computing unit operation rate, storage space size, cost, etc., for allocation and scheduling by the blockchain.

In the second step, the blockchain authenticates the applied node and integrates the resources provided by the node to establish a virtual resource pool. The blockchain also maintains a virtual resource status table, which records the allocation and usage of resources, the mapping of virtual ports to physical addresses, and so on.

In the third and fourth steps, the participant node applies to instantiate a federated learning training task, and the blockchain selects the training node according to the data description information provided during the registration of each training node.

In the 5th step, each participant node negotiates the relevant parameters of the federated learning training task, including the number of training rounds, the initialization model, the total budget cost, etc., and uses digital signature technology to sign a smart contract.

In the 6th step, the smart contract of blockchain instantiates a federated learning task, determines the initialization priority of the task, saves its initial state on the chain, and places it in the task queue to wait for the allocation and scheduling of computing resources.

In the 7th step, the blockchain reasonably allocates the federated learning tasks in the waiting state to different virtual computing units. The blockchain obtains the physical address of the virtual resource by searching the resource state table and then encrypts it, establishing the trusted computing sandbox as the state channel and updating the resource usage state. The participant node only knows the information of the allocated virtual resources, and it cannot obtain the real physical address of the computational sandbox, so it cannot carry out malicious attacks on the model in training.

In the 8th to 12th steps, participants perform specific federated learning training. The smart contract initializes the model and sends it to each training node. Then, the nodes update the local model with local data and upload the gradient and other parameter information to the state channel for global model aggregation. After the aggregation is completed, the updated global model is sent to each training node for a new round of training until the training is completed. In the training process, the behavior and status of each node are supervised by all nodes. Once malicious behavior is found, it is immediately reported to the blockchain and the malicious node is punished accordingly.

In the 13th to 15th steps, after completing the federated learning training task, the training node updates the final results and status information to the blockchain and then immediately releases the occupied resources, closing the state channel.

In the 16th and 17th steps, the blockchain sends the final result to the task requester, updates the status information of virtual resources, and waits for the arrival of new tasks for resource allocation and scheduling.

In the following sections, we will focus on resource scheduling during the establishment of the state channel and discuss how to allocate resources to make the whole system work more efficiently while satisfying the requirements of tasks on service quality as much as possible.

## 4. Resource Scheduling Problem Modeling

In the process of constructing state channels for federated learning tasks, different resource scheduling schemes may affect the working efficiency of the system and the quality of completion of tasks. Improper scheduling policies may cause the computing load of some nodes to be too heavy and cause faults, while the resource utilization of other nodes decreases, resulting in resource waste and greatly reducing system efficiency. There may also be unreasonable resource allocation schemes that make it difficult to meet the requirements of tasks on service quality, such as exceeding the budget and the expected completion time. In this section, a model of the resource-scheduling optimization problem under the state channel-based federated learning training supervision mechanism is constructed.

In this paper, the resource scheduling problem is defined as how to assign multiple federated tasks to multiple computing nodes, such as abnormal behavior detection task, risk assessment task, customer behavior analysis task, product intelligence recommendation task, etc., so as to obtain a scheduling scheme that minimizes the completion time of the whole system under the constraints of task cost and task completion time.

We assume that the training node set of the participants is denoted as $Nodes=\{N_{1},N_{2},\ldots ,N_{N_{node}}\}$, where $N_{i}$ indicates the node i and $N_{node}$ indicates the number of nodes. Nodes are represented as $N_{i}=\left\{PU_{1},PU_{2},\ldots ,PU_{N_{pu}^{i}}\right\}$, where $PU_{j}\left(j=1,2,\ldots ,N_{pu}\right)$ represents the j-th processing unit, and $N_{pu}^{i}$ represents the number of processing units of the i-th node. The processing unit is represented as PU={SIDP,E,COST,TD}, where SIDP is the number of the processing unit, *E* is the execution capacity of the processing unit, COST is the cost that needs to be paid for using the processing unit to execute the unit time, and TD is the communication delay between the node where PU is located and other nodes. $TD_{i,j}$ indicates the communication delay from node i to node j.

We assume that the federated learning task set is expressed as $Tasks=\{T_{1},T_{2},\ldots ,T_{N_{task}}\}$, where $T_{i}$ represents task i and $N_{task}$ represents the number of tasks. Tasks are represented as $T=\{SIDT,WorkLoad,MaxT,MaxC,PI,N_{train}\}$, where SIDT is the serial number of the learning task, WorkLoad is on behalf of its quota, MaxT represents the maximum completion time a task can bear, and MaxC represents the maximum cost a task can bear. In this paper, MaxT and MaxC are taken as indicators of the requirements of tasks on service quality, PI represents the priority of the task, and $N_{train}$ represents the set of training nodes participating in the task.

Therefore, we can use a matrix to represent the task resource allocation scheme. The allocation matrix is defined as follows:

(1) $$X=\left[\begin{array}{cccc} x_{1,1} & x_{1,2} & \ldots & x_{1,N_{pu}}\\ x_{2,1} & x_{2,2} & \ldots & x_{2,N_{pu}}\\ \ldots & \ldots & \ldots & \ldots \\ x_{N_{task},1} & x_{N_{task},2} & \ldots & x_{N_{task}},N_{pu} \end{array}\right]$$

where *X* is the task resource allocation matrix with the size of $N_{task}*N_{pu}$, and $x_{i,j}$ represents the assignment of the i-th task on the j-th block processing unit, which is defined as

(2) $$x_{i,j}=\left\{\begin{array}{c} 1,\;\mathrm{if}\;T_{i}\;\mathrm{is}\;\mathrm{allocated}\;\mathrm{to}\;PU_{j}\\ 0,\;\mathrm{if}\;T_{i}\;\mathrm{is}\;\mathrm{not}\;\mathrm{allocated}\;\mathrm{to}\;PU_{j} \end{array}\right.$$

According to the task resource allocation matrix, we can calculate the completion time of each task. The calculation formula is as follows:

(3) $$ECT_{i}=\frac{WorkLoad_{i}}{\sum_{j=1}^{Npu}x_{i,j^{*}}E_{j}}$$

Similarly, the cost of each task can be calculated by the following formula:

(4) $$TCost_{i}=\sum_{j=1}^{N_{pu}}ECT_{i}*COST_{j}*x_{i,j}$$

We can also calculate the maximum completion time of the system by the following formula:

(5) $$=MAX_{j\in \left[1,N_{pu}\right]}\left\{\sum_{i=1}^{N_{task}}ECT_{i}*x_{i,j}\right\}$$

Finally, resource scheduling is abstracted as a goal optimization problem, that is, solving the resource allocation matrix X meets the following conditions:

(6) $$\begin{array}{c} \mathrm{Minimize}\;MAX_{j\in \left[1,N_{pu}\right]}\left\{\sum_{i=1}^{N_{task}}ECT_{i}*x_{i,j}\right\}\\ \;s.t.\;\forall i\in \left[1,N_{task}\right],ECT_{i}\leq MaxT_{i}\;\mathrm{and}\;TCost_{i}\leq MaxC_{i} \end{array}$$

The specific system parameters in this section are shown in Table 1.

## 5. DRL-Based Resource Scheduling Algorithm

### 5.1. Algorithm Framework and Mechanism

**Figure 3:**
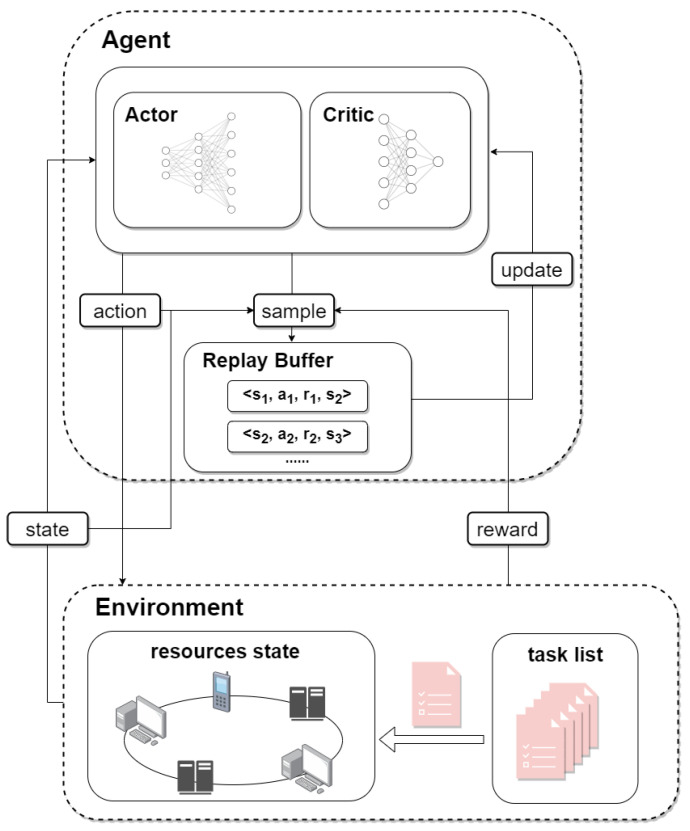
Resource-scheduling algorithm framework based on DRL.

In the complex federated learning training environment based on blockchain, the resource-scheduling algorithm needs to select the optimal resource allocation scheme according to the current node resource state and the federated learning task waiting for resource allocation. In this paper, an Actor–Critic resource-scheduling algorithm based on DRL has been designed. The algorithm framework is shown in Figure 3, which is mainly divided into two parts, environment and agent.

The environment is responsible for monitoring the state of each node, managing the computing resources they own, and maintaining a list of tasks awaiting resource allocation. The environment provides the current state information to the agent, including the state of the current resource and the state of the task to be assigned, and it returns the reward of the current operation and the state of the next time according to the actions made by the agent.

The agent is a resource scheduler, making decisions and choosing actions based on the state given by the environment. The system allocates processing resources to tasks based on actions. The agent is composed of the Critic value network and the Actor policy network. The Critic network can score and evaluate the current state, while the Actor network selects the current optimal action according to the environment state.

Traditional Q learning has some disadvantages such as a waste of previous experience and correlation of parameter updating. This paper adopted the method of experience replay, set the experience buffer pool R, and stored the environmental state, the action performed, the reward received, and the state of the next moment as experience samples in the buffer pool. When training network parameters, small batch data can be randomly extracted for training and parameter update so as to make better use of the previous experience and break the correlation of parameter update.

Before resource scheduling, the system sorts the tasks waiting for resource allocation based on their priorities and then allocates resources to them. Suppose that the priority of each task is denoted as $Priorities=\{PI_{1},PI_{2},\ldots ,PI_{N_{task}}\}$, where $PI_{i}$ represents the priority of task i and it is represented as $PI_{i}=initialPr_{i}+waitTime_{i}$, where $initialPr_{i}$ is the initial priority of the task and related to the $WorkLoad_{i}$. The lower the workload, the higher the priority, which takes advantage of the short-job-first strategy and helps to reduce the average wait time. $waitTime_{i}$ is the time the ith task is waiting for resource allocation, which is a dynamic value. The longer the waiting time, the higher the priority, which is conducive to reducing the problem of operation hunger.

When training the Actor–Critic algorithm network, the system makes an action $a_{t}$ based on the policy network according to the current environment state $s_{t}$. Then, the reward $r_{t}$ is obtained by interacting with the environment, and the new environment state $s_{t+1}$ is obtained, and then, $\left(s_{t},a_{t},r_{t},s_{t+1}\right)$ is put into the buffer pool *R* as a sample. Finally, *N* groups of data samples were randomly selected from the buffer pool, and the Actor–Critic network parameters were updated by calculating the average value of the gradient.

#### 5.1.1. Train Critic Value Network

Define the discount return $U_{t}$ as follows:

(7) $$U_{t}=r_{t}+\gamma r_{t+1}+\gamma^{2}r_{t+2}+\cdots +\gamma^{n}r_{t+n}$$

where $U_{t}$ represents the cumulative reward since time *t*, $r_{t}$ represents the reward at time *t*, γ∈(0,1) is the parameter, and the subsequent reward should have a lower weight than the current reward.

In order to judge whether an action is good or bad in the current state, the action value function is defined as follows:

(8) $$Q_{\pi }\left(s_{t},a_{t}\right)=E\left[U_{t}|S_{t}=s_{t},A_{t}=a_{t}\right]$$

where $Q_{\pi }\left(s_{t},a_{t}\right)$ represents the expectation of discount return $U_{t}$, and $s_{t}$ and $a_{t}$ are the state at time *t* and the actions made under this state respectively.

In order to further judge the quality of a certain state, we define the state value function as follows:

(9) $$V_{\pi }\left(s_{t}\right)=E_{A}\left[Q_{\pi }\left(s_{t},A\right)\right]$$

where $V_{\pi }\left(s_{t}\right)$ represents the expectation of action value function to action *A*, namely the score of the current state $s_{t}$. In this paper, a neural network v(s;w) is used to approximate the state value function. The purpose of the training value network is to make the system score the state more and more accurately, closer to the return given by the real environment.

In this paper, we use the sum of the reward given by the current action and the rating of the state at the next time as an estimate of the true reward, which is defined as follows:

(10) $$y_{t}=r_{t}+\gamma v\left(s_{t+1};w\right)$$

where $y_{t}$ is the estimate of $U_{t}$ and the target value for training the neural network. Therefore, the loss function can be expressed as:

(11) $$loss=\frac{1}{2}{\left(v\left(s_{t};w\right)-y_{t}\right)}^{2}$$

So, the gradient of the value network is:

(12) $$g_{w}=\delta_{t}\cdot \frac{\partial v\left(s_{t};w\right)}{\partial w}$$

where $\delta_{t}$ is represented as:

(13) $$\delta_{t}=v\left(s_{t};w\right)-y_{t}$$

Since $v(s_{t};w)$ is not the actual observed return, but the estimate of the real discount return, which is often prone to bias. Updating the value network parameters through bootstrapping is prone to overestimation, which is aggravated by continuing to use the already overestimated network to predict the next training values. Therefore, we use target value network $v(s_{t};w^{\prime })$ for estimation. The $v(s_{t};w^{\prime })$ and $v(s_{t};w)$ have the same structure, and the same initial value, but different updating methods. Each time $y_{t}$ is calculated, the $v(s_{t};w^{\prime })$ is used for estimation. The calculation formula is as follows.

(14) $$y_{t}=r_{t}+\gamma v\left(s_{t+1};w^{\prime }\right)$$

Other calculations remain the same, and the following formula is used when $w^{\prime }$ is updated.

(15) $$w^{\prime }\leftarrow \tau \cdot w+\left(1-\tau \right)\cdot w^{\prime }$$

where τ∈(0,1) is the parameter.

#### 5.1.2. Train Actor Policy Network

The policy function π(a∣s) can calculate the probability distribution of the action according to the current state, which is used to make decisions according to the environmental state. This paper uses a neural network π(a∣s;θ) to approximate the policy function. The goal of training strategy networks is to obtain a bigger discount on the output of actions in the face of different state inputs.

The traditional calculation method of policy gradient is:

(16) $$g_{\theta }=\nabla_{\theta }\log\pi \left(a_{t}|s_{t};\theta \right)U_{t}$$

In order to improve the convergence efficiency of reinforcement learning algorithm, this paper adopts the policy gradient calculation method based on a baseline, and the gradient calculation formula becomes:

(17) $$g_{\theta }=\nabla_{\theta }\log\pi \left(a_{t}|s_{t};\theta \right)\left[U_{t}-b\right]$$

where *b* is the parameter independent of action $a_{t}$. By subtracting b from $U_{t}$, the variance of the gradient is reduced to accelerate the convergence rate of the algorithm. For *b*, we choose the state value network $v(s_{t};w)$ and use Equation (14) to estimate $U_{t}$, and we finally obtain the calculation method of policy gradient based on the baseline:

(18) $$g_{\theta }=-\nabla_{\theta }\log\pi \left(a_{t}|s_{t};\theta \right)\delta_{t}$$

### 5.2. MDP Model

To use the deep reinforcement learning method to solve the resource-scheduling optimization problem proposed in Section 4, we expressed the problem as an MDP model, which mainly includes state space, action space, and reward. The specific design is as follows.

The state space should include the allocation of each processing resource at time t and the specific state of the task to be allocated at time *t*, such as the workload, budget, and the maximum completion time of the task that can be tolerated. Therefore, the state space is defined as:

(19) $$s_{t}=\left\{X_{t},WorkLoad_{i},MaxT_{i},MaxC_{i}\right\}$$

where $WorkLoad_{i}$, $MaxT_{i}$ and $MaxC_{i}$, respectively, represent the workload, maximum completion time that can be tolerated, and the maximum budget cost of the *i*-th task.

The action space represents the corresponding actions that the agent can perform in the state $s_{t}$. The system will remove the task with the highest priority from the wait queue and allocate resources to it. The actions are defined as follows:

(20) $$a_{t}=\left\{\gamma_{1},\gamma_{2},\ldots ,\gamma_{N_{pu}}\right\}$$

where $\gamma_{i}\in \left\{0,1\right\}$ indicates how the task is allocated on the *i*-th resource: 0 for unallocated and 1 for allocated.

The reward is the score that the agent obtains after taking an action based on the current state of the environment to evaluate the goodness of the action. The system calculates the reward of the action according to whether the requirements of tasks on service quality are satisfied after resource allocation and the completion time of the system. With the maximum completion time of the system as the standard of reward, if the action of resource allocation causes the completion time and cost of the task to be greater than the maximum that the task can endure, there will be a huge penalty. The reward can be defined as:

(21) $$r_{t}=\left\{\begin{array}{c} -5,ECT_{i}>MaxT_{i}\;\mathrm{or}\;Tcost_{i}>MaxC_{i}\\ -\varepsilon \left(maxMakespan_{t}-maxMakespan_{t-1}\right),\;\mathrm{else}\; \end{array}\right.$$

where ε is the system parameter.

### 5.3. Proposed Algorithm

The specific steps of the algorithm are shown in Algorithm 1. The algorithm is trained for *M* epochs, and the network parameters are updated *T* times in each epoch. At the beginning of each epoch, a task waiting queue is randomly initialized, and a random initial state is obtained by observing the environment. Before each parameter update, the system obtains a set of $\left(s_{t},a_{t},r_{t},s_{t+1}\right)$ samples from the environment and puts them into the buffer pool *R*. When the amount of data in *R* reaches the set value, *N* groups of samples are randomly selected from *R* for batch gradient update of network parameters.
**Algorithm 1** A resource-scheduling algorithm based on Actor–Critic.**Input:** Task queues;**Output:** A resource-scheduling model;1:Initialize the Actor network π(a∣s;θ).2:Initialize the Critic network $v(s_{t};w)$ and target value network $v(s_{t};w^{\prime })$.3:Initialize the experience replay buffer pool *R*.4:**for all**j=1,2,⋯,M**do**5: Randomly initialize a task-waiting queue and observe an initial random state $s_{t}$.6: **for all** i=1,2,⋯,T7:  Select an action $a_{t}$ based on the Actor network $\pi (a_{t}|s_{t};\theta )$.8:  Obtain the environment state of the next moment $s_{t+1}$ and calculate the corresponding reward of the action $a_{t}$ according to Equation 21.9:  The multiple sets of samples $\left(s_{t},a_{t},r_{t},s_{t+1}\right)$ obtained by repeating the above process several times are put into the experience replay buffer pool *R*.10:  If the amount of data in *R* reaches the set value, *N* batches of samples $\left(s_{i},a_{i},r_{i},s_{i+1}\right)$ are randomly selected from R to train the parameters in the neural network.11:  Calculate $y_{i}$ according to Equation 14.12:  Calculate $\delta_{i}$ according to Equation 13.13:  Calculate the policy network gradient: $g_{\theta }=-\frac{1}{N}\sum_{i}\nabla_{\theta }\log\pi \left(a_{i}|s_{i};\theta \right)\delta_{i}$14:  Calculate the value network gradient: $g_{w}=\frac{1}{N}\sum_{i}\delta_{i}\cdot \frac{\partial v\left(s_{i};w\right)}{\partial w}$15:  Update policy network: $\theta \leftarrow \theta +\beta \cdot g_{\theta }$16:  Update value network: $w\leftarrow w-\alpha \cdot g_{w}$17:  After iteration *k* times, update the target value network according to Equation 15.18: **end for**19:**end for**

In the gradient update process, $y_{i}$ and $\delta_{i}$ are calculated using Equations (13) and (14). Then, the gradient of the policy network is calculated by weighted average according to Equation (18). $y_{i}$ served as the target value label of the value network, and the gradient of the value network is calculated by weighted average according to Equation (12). The calculated gradient is used to update the parameters of the policy network and value network, respectively. Finally, the parameters of the target value network are updated after iteration *k* times.

## 6. Experiments and Results

### 6.1. Experimental Settings

To simulate and verify the effectiveness of the proposed deep reinforcement learning algorithm in solving resource-scheduling problems, this paper uses the Gym framework of OpenAI to model the environment involved in resource-scheduling problems, and it uses the open source framework Tianshou [34] based on PyTorch to conduct simulation experiments on the proposed algorithm. The experiment simulates a scenario in which the system allocates and schedules incoming tasks according to existing computing resources in the process of constructing the state channel in the blockchain environment. The scenario consists of 10 processing units and 100–1000 federated learning tasks with different requirements on service quality. Each unit has a computation rate of 500–2000 MIPS and an overhead of 5–20 per unit of time. The workload length of each task is 100–4000 MI, the budget is 10–50, and the maximum completion time of the task that can be tolerated is 5–10 s. The setting of specific experimental parameters is shown in Table 2.

The Actor–Critic algorithm has 100 epochs, and 5000 updates of network parameters are performed in each epoch. The buffer size is set to 2000, and the learning rate of both the Actor network and Critic network is 1 × 10^−4^. The specific algorithm parameter settings are shown in Table 3.

In this paper, several algorithms are selected to compare with the proposed algorithm (A2C). (1) In the random selection algorithm, each federated learning task is randomly allocated a block of computing resources. (2) The greedy algorithm only considers scheduling schemes that can minimize the maximum completion time of the system. (3) Genetic algorithm (GA) [21] is designed and proposed according to the biological evolution law in nature and simulates the natural selection and biological evolution process, which is a common method to solve the problem of objective optimization.

### 6.2. Analysis of Results

In this paper, several evaluation indicators of the algorithm are set as follows:

- maxMakespan, the maximum completion time of the system, is used to evaluate the efficiency of the system, as defined in Equation (5).
- avgCost, the average task cost, which evaluates the cost of a task, is defined as follows:
  (22) $$avgCost=\frac{1}{N_{task}}\sum_{i=1}^{N_{task}}\sum_{j=1}^{N_{pu}}ECT_{i}*COST_{j}*x_{i,j}$$
- avgCompletedTime, the average task competition time, is defined as follow:
  (23) $$avgCompletedTime=\frac{1}{N_{task}}*\sum_{i=1}^{N_{task}}\frac{WorkLoad_{i}}{\sum_{j=1}^{Npu}x_{i,j}*E_{j}}$$
- mSLA, the percentage of the tasks that meet the requirements on service quality in the total tasks, evaluates the ability of the algorithm to meet the requirements of tasks on service quality, which is defined as follows:
  (24) $$mSLA=\frac{N_{mSLA}}{N_{task}}$$
  where $N_{mSLA}$ represents the proportion of tasks that meet the requirements on service quality among all tasks. This paper assumes that the indicators of the requirements of tasks on service quality are TCost and ECT; that is, the tasks that meet the two indicators are defined as the tasks that meet the quality of service requirements.

**Figure 4:**
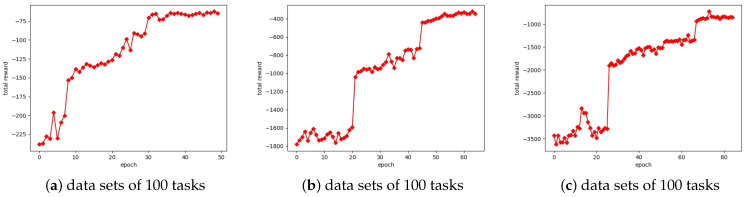
The total reward of the A2C on data sets of different numbers of tasks.

Figure 4 shows the change of the total reward of the Actor–Critic algorithm on 100, 500, and 1000 test tasks set, respectively. As can be seen from the figure, the total reward of the model obtained at the initial stage of training is low on the task set, which indicates that the model needs to be trained a certain number of times to obtain a better resource allocation scheme. In addition, it can be found that the convergence rate of the algorithm on the different number of tasks is not the same. In general, the more tasks the algorithm converges, the slower the algorithm converges. When the number of training iterations reaches 40, 50, and 70, the total reward of the algorithm on 100, 500, and 1000 test tasks set gradually becomes stable.

This paper illustrates the advantages of DRL in resource scheduling by comparing it with other three heuristic and meta-heuristic resource-scheduling algorithms. Table 4 shows the performance of four different resource-scheduling algorithms on the different number of task sets. The indexes are maxMakespan (the maximum completion time of the system), avgCost (the average task cost, which evaluates the cost of a task), avgCompletedTime (the average task competition time), and mSLA (the proportion of tasks that meet the requirements on service quality among all tasks). The four algorithms are the random selection algorithm, greedy algorithm, GA (genetic algorithm), and A2C (the proposed algorithm based on deep reinforcement learning).

**Figure 5:**
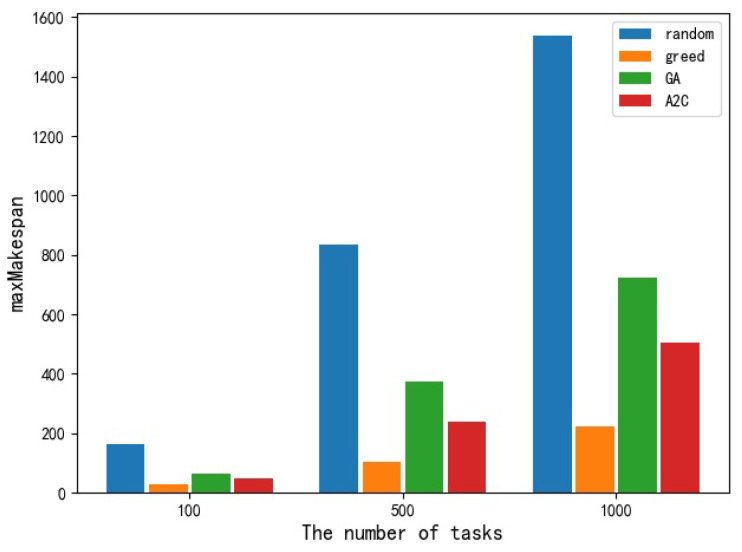
Comparison of different algorithms in maxMakespan.

As for the performance of the maxMakespan index, it can be seen from the table that the maximum completion time of the system obtained by using a random scheduling algorithm on 100, 500, and 1000 task sets is the largest among the four scheduling algorithms and is proportional to the number of tasks. The GA algorithm and A2C algorithm come second, and the greedy strategy has the least. This trend also increases with the number of tasks, and the gap between different algorithms becomes more and more obvious. Figure 5 shows the histogram of different scheduling algorithms on maxMakespan, which can intuitively show the advantages and disadvantages of different algorithms on maxMakespan.

**Figure 6:**
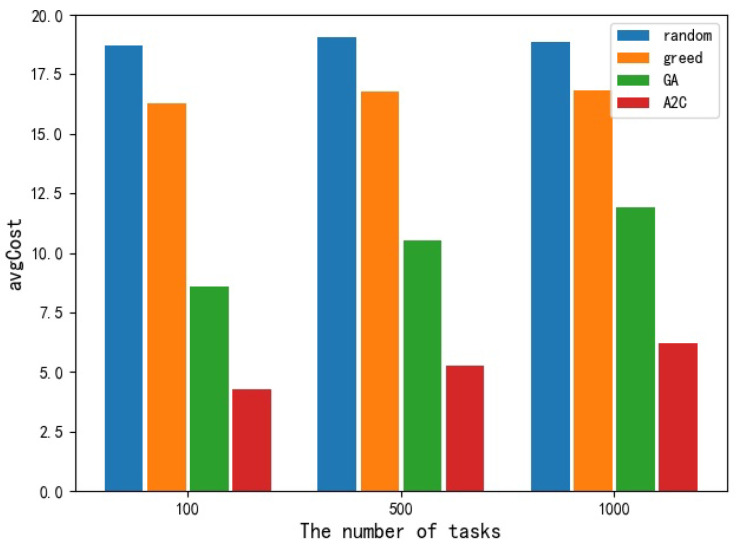
Comparison of different algorithms in avgCost.

**Figure 7:**
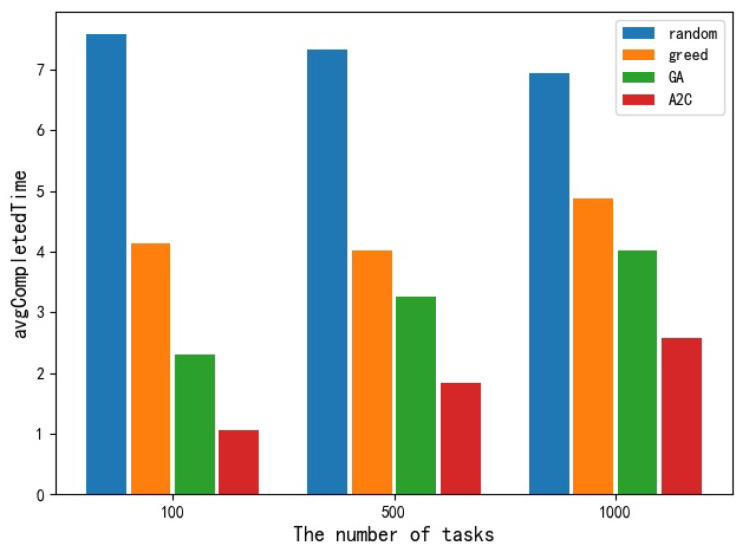
Comparison of different algorithms in avgCompletedTime.

For avgCost and avgCompletedTime, different from maxMakespan, the greedy algorithm performed poorly in these two indexes. The avgCost obtained by the greedy algorithm is only slightly better than that of the random selection scheduling algorithm, but the gap is not big. The performance of the other three algorithms on this index from bad to good is the random selection algorithm, GA algorithm, and A2C algorithm, respectively. Figure 6 and Figure 7, respectively, show the histogram of performance of different scheduling algorithms at avgCost and avgCompletedTime.

**Figure 8:**
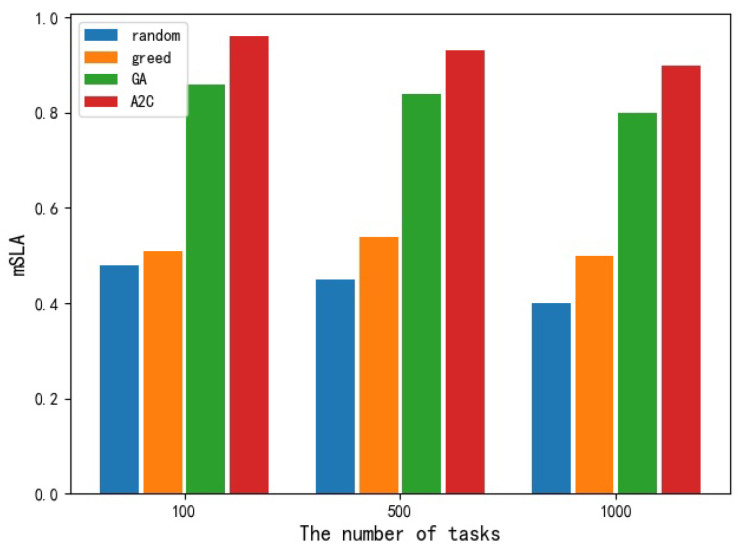
Comparison of different algorithms in mSLA.

As for the mSLA index, it can be seen that the random selecting scheduling algorithm on the test set can only ensure that less than 50% of the tasks meet the requirements of service quality. The greedy algorithm and random selection algorithm are not much different. The GA algorithm can ensure that about 80% of the tasks meet the quality of service requirements. The A2C algorithm can ensure that more than 90% of the tasks can meet the requirements under different task numbers, but the proportion decreases with the increase of tasks. Figure 8 shows the histogram of the performance of different resource-scheduling algorithms on mSLA.

Combined with the pictures and the table, random selection scheduling has the worst effect on any index because of its randomness and uncertainty. Although the greedy algorithm has an excellent performance in the completion time of the system, it can not well meet the service quality requirements of the task, and the effect of other evaluation indicators is not good. This is because the greedy algorithm only considers the maximum completion time of the system and does not take into account other factors such as the task’s quality of service requirements. The A2C algorithm and GA algorithm not only consider the maximum completion time of the system but also consider the task quality of service requirements and other constraints. The evaluation on the different number of tasks and different indexes shows that the A2C algorithm has a better effect than the traditional GA algorithm on this problem model. For different system sizes and complex environments, the A2C algorithm based on deep reinforcement learning can constantly optimize its scheduling strategy through the feedback of the environment, and after a certain degree of pre-training, it can conduct efficient and fast scheduling. Experiments show that the proposed algorithm can reduce the maximum completion time of the system based on satisfying the requirements of task quality of service, which makes the whole blockchain system more efficient in scheduling federated learning and training tasks.

## 7. Conclusions

This paper designs a federated learning multi-task scheduling mechanism based on a trusted computing sandbox. First of all, a system framework for completing federated learning tasks in the blockchain environment is proposed. The blockchain reasonably allocates resources for each federated learning task and constructs a computing sandbox as a state channel. The federated learning model training process is carried out in the channel and supervised by all nodes. Secondly, considering the factors such as the resource heterogeneity of each participating node, system efficiency, and the requirements of tasks on service quality, an optimization problem model of resource allocation and scheduling in the blockchain scenario was constructed. Finally, the problem model was constructed as an MDP model, and a resource-scheduling algorithm based on Actor–Critic was designed to solve the problem. The experimental results show that the proposed algorithm has good convergence under different task number scenarios, and it can reduce the maximum completion time of the system and improve the efficiency of the system while meeting the requirements of tasks on service quality.

For future work, the privacy and security issues behind the value sharing of a large amount of data will be paid more and more attention. Federated learning and blockchain technology will play an increasingly important role in the field of data privacy and security through the advantages of local data and the efficient and safe data-sharing method of decentralization. How to protect user data privacy through federated learning in the decentralized mode will become the focus of future research. In the next step, we will study how to realize the discovery and supervision of malicious behaviors of nodes in the computing sandbox during the training of federated learning to prevent privacy leakage and attacks in time.

## Figures and Tables

**Table 1:** System Parameters.

Parameters	Meaning
Nodes	Set of participant nodes
$N_{node}$	Number of participating nodes
PU	Processing unit
$N_{pu}$	Number of processing units
SIDP	The serial number of processing unit
*E*	The execution capacity of a processing unit in instructions per second
COST	The execution cost per unit of time of the processing unit
$TD_{i,j}$	Communication delay from node i to node j
Tasks	Federated learning task set
$N_{task}$	The number of tasks
SIDT	The serial number of the task
WorkLoad	The workload of a task, expressed as the number of instructions
MaxT	The maximum completion time the task can tolerate
MaxC	The maximum cost that the task can bear
PI	The priority of the task
$N_{train}$	Set of training nodes for the task
$x_{i,j}$	The distribution of the ith task on the jth processing unit
$ECT_{i}$	Completion time of task i
$TCost_{i}$	The cost of task i
maxMakespan	Maximum system completion time

**Table 2:** Experimental Environment Parameters.

Parameters	Settings
NUMBER OF COMPUTING UNITS	10
COMPUTATION RATE	500–2000 MIPS
COST WITHIN A UNIT OF TIME	5–20
NUMBER OF TASKS	100–1000
TASK LENGTH	100–4000 MI
TASK BUDGET	10–50
MAXIMUM COMPLETION TIME OF TASK	5–10 s

**Table 3:** Parameters of A2C Algorithm.

Parameters	Settings
Epoch (*M*)	100
Step per epoch (*T*)	50,000
Buffer size (*S*)	2000
Batch size (*N*)	64
γ	0.9
*k*	100
α	1–e4
β	1–e4
τ	0.5
ε	1

**Table 4:** The performance of the four scheduling algorithms on each metric. The columns give the results of the algorithms on the different evaluation metrics and the rows indicate the different scheduling algorithms. Each metric is further divided into results on sets of 100, 500, and 1000 number of tasks.

Scheduling Algorithms	maxMakespan			avgCost			avgCompletedTime			mSLA		
	100	500	1000	100	500	1000	100	500	100	100	500	1000
Random	164	833	1537	7.58	7.34	6.95	18.71	19.05	18.85	0.48	0.45	0.44
Greed	27	103	224	4.14	4.02	4.88	16.3	16.79	16.83	0.51	0.54	0.55
GA	62	374.2	722	2.3	3.25	4.01	8.6	10.54	11.93	0.86	0.84	0.82
A2C	47	237	503	1.05	1.83	2.57	4.27	5.28	6.2	0.96	0.93	0.91
